# Interchromosomal template-switching as a novel molecular mechanism for imprinting perturbations associated with Temple syndrome

**DOI:** 10.1186/s13073-019-0633-y

**Published:** 2019-04-23

**Authors:** Claudia M. B. Carvalho, Zeynep Coban-Akdemir, Hadia Hijazi, Bo Yuan, Matthew Pendleton, Eoghan Harrington, John Beaulaurier, Sissel Juul, Daniel J. Turner, Rupa S. Kanchi, Shalini N. Jhangiani, Donna M. Muzny, Richard A. Gibbs, Baylor-Hopkins Center for Mendelian Genomics, Pawel Stankiewicz, John W. Belmont, Chad A. Shaw, Sau Wai Cheung, Neil A. Hanchard, V. Reid Sutton, Patricia I. Bader, James R. Lupski

**Affiliations:** 1 Department of Molecular and Human Genetics, Baylor College of Medicine, One Baylor Plaza, Room 604B, Houston, TX 77030-3498 USA; 2 Oxford Nanopore Technologies Inc, New York, NY USA; 3 Oxford Nanopore Technologies Inc, San Francisco, CA USA; 4 Oxford Nanopore Technologies Ltd, Oxford, UK; 5 UT MD Anderson Cancer Center, Houston, TX USA; 6 Human Genome Sequencing Center, Baylor College of Medicine, Houston, TX 77030 USA; 7 Department of Pediatrics, Baylor College of Medicine, Houston, TX 77030 USA; 8 Texas Children’s Hospital, Houston, TX USA; 9 Carle Clinic, Urbana, IL 61801 USA

**Keywords:** Triplication, DUP-TRP/INV-DUP, Complex genomic rearrangement, MMBIR, Replicative-based mechanism, Inter-homologous chromosomal template switch, Runs of homozygosity (ROH), ES, Absence of heterozygosity (AOH)

## Abstract

**Background:**

Intrachromosomal triplications (TRP) can contribute to disease etiology via gene dosage effects, gene disruption, position effects, or fusion gene formation. Recently, post-zygotic de novo triplications adjacent to copy-number neutral genomic intervals with runs of homozygosity (ROH) have been shown to result in uniparental isodisomy (UPD). The genomic structure of these complex genomic rearrangements (CGRs) shows a consistent pattern of an inverted triplication flanked by duplications (DUP-TRP/INV-DUP) formed by an iterative DNA replisome template-switching mechanism during replicative repair of a single-ended, double-stranded DNA (seDNA), the ROH results from an interhomolog or nonsister chromatid template switch. It has been postulated that these CGRs may lead to genetic abnormalities in carriers due to dosage-sensitive genes mapping within the copy-number variant regions, homozygosity for alleles at a locus causing an autosomal recessive (AR) disease trait within the ROH region, or imprinting-associated diseases.

**Methods:**

Here, we report a family wherein the affected subject carries a de novo 2.2-Mb TRP followed by 42.2 Mb of ROH and manifests clinical features overlapping with those observed in association with chromosome 14 maternal UPD (UPD(14)mat). UPD(14)mat can cause clinical phenotypic features enabling a diagnosis of Temple syndrome. This CGR was then molecularly characterized by high-density custom aCGH, genome-wide single-nucleotide polymorphism (SNP) and methylation arrays, exome sequencing (ES), and the Oxford Nanopore long-read sequencing technology.

**Results:**

We confirmed the postulated DUP-TRP/INV-DUP structure by multiple orthogonal genomic technologies in the proband. The methylation status of known differentially methylated regions (DMRs) on chromosome 14 revealed that the subject shows the typical methylation pattern of UPD(14)mat. Consistent with these molecular findings, the clinical features overlap with those observed in Temple syndrome, including speech delay.

**Conclusions:**

These data provide experimental evidence that, in humans, triplication can lead to segmental UPD and imprinting disease. Importantly, genotype/phenotype analyses further reveal how a post-zygotically generated complex structural variant, resulting from a replication-based mutational mechanism, contributes to expanding the clinical phenotype of known genetic syndromes. Mechanistically, such events can distort transmission genetics resulting in homozygosity at a locus for which only one parent is a carrier as well as cause imprinting diseases.

**Electronic supplementary material:**

The online version of this article (10.1186/s13073-019-0633-y) contains supplementary material, which is available to authorized users.

## Background

Intrachromosomal triplications (TRP) are defined as a heterozygous segmental 3X copy-number amplification of a given locus in which all three copies are inserted in cis. For a TRP locus, the total number of alleles will be equivalent to four copies for autosomes and equal to three copies for the X-chromosome of 46, XY males [1]. TRP can potentially contribute to disease etiology via gene dosage effects [2, 3], gene disruption at breakpoint junctions [4], position effects [5, 6], or fusion gene formation [7]. Triplication of dosage-sensitive genes can increase phenotypic severity in triplication versus duplication carriers [2, 3, 8–11], lower the disease age-of-onset [12], or result in distinct clinical phenotypes [13] compared to duplications affecting the same locus. Moreover, locus duplication increases the risk of triplication formation as a result of unequal crossover (i.e., nonallelic homologous recombination; NAHR) by about 100X [11, 14, 15]; such type of rearrangement within a given locus can result in altered inter-generational disease penetrance [16–18] with the appearance of genetic anticipation [11].

The contribution and impact of triplication on disease etiology is still under-recognized [19] due, in part, to the technical limitations in distinguishing duplications from triplications or higher order (e.g. quadruplication and further amplification) copy-number gains [11, 18]; this molecular diagnostic challenge may result in triplications being miscalled as duplications. We and others have reported a rare type of de novo complex genomic rearrangement (CGR) constituted by TRP followed by absence of heterozygosity (AOH) in patients clinically referred for multiple congenital abnormalities [20–24]. TRP segments are inverted and can be as large as 21.7 Mb in size in addition to being flanked by small duplications of a few Kb, a structure named inverted triplication flanked by duplications (DUP-TRP/INV-DUP) [10, 22]. The copy-number neutral AOH segment can encompass > 50 Mb extending to the telomere and reflects segmental uniparental isodisomy (UPD) whereas the triplications are formed by biparental contributions [22], and both variant types are potentially generated concomitantly in a post-zygotic event [22]. Genotype-phenotype correlations suggest that clinical abnormalities result from gene dosage and expression alterations of genes included within the copy-number gain interval(s) [24] and from unmasking of biallelic autosomal recessive (AR) disease traits; as an example of the latter, Sahoo and colleagues reported a patient with autosomal recessive citrullinemia (MIM#215700) in whom a paternal UPD segment included a paternally inherited pathogenic variant in *ASS1* [23]. Moreover, some of the subjects with TRP/AOH may also present clinical phenotypes due to the inheritance of an imprinted locus within the segmental UPD, which was predicted based on the underlying formation mechanism but not yet reported in patients with genomic disorders [25].

Here, we report an individual proband and family in which family-based genomic analyses revealed a de novo 2.2-Mb TRP followed by 42.2 Mb of AOH involving chromosome 14 in the proband. Segregation analysis of single-nucleotide polymorphism (SNP) array data indicates that the copy-number neutral runs of homozygosity (ROH) segment results from maternal uniparental isodisomy for chromosome 14 (UPD(14)mat) [26], a molecular diagnosis which may lead to a clinical diagnosis of Temple syndrome [MIM#616222]. Further assessment of differentially methylated regions (DMR) within the *DLK1*-*DIO3* locus, using independent genomic approaches of genome-wide methylation arrays and nanopore sequencing, confirmed the typical methylation pattern observed in UPD(14)mat patients; the index case indeed presents clinical features overlapping those observed with Temple syndrome. In conclusion, our data provide experimental evidence that, in humans, post-zygotically generated in cis triplications can also present with segmental UPD in association with altered methylation and imprinting-associated disease due to the loss of one parental contribution at a locus. This further increases the potential genomic and pathogenetic consequences of this type of structural variant mutagenesis event and resultant CGR.

## Methods

### Subjects

Index subject, BAB7004, carrying the triplication associated with AOH was identified by the Baylor Genetics clinical diagnostic laboratory by chromosome microarray analysis (CMA) [27, 28] as part of their clinical evaluation. Informed consent was obtained for participation in further genome-wide research studies of all family members, protocols no. H-25466 and/or no. H-29697, both approved by the institutional review board at Baylor College of Medicine. Genomic DNA from the index patient, BAB7004, and unaffected family members (family pedigree HOU2583): BAB7088 (father), BAB7291 (mother), and siblings, BAB7085, BAB7086, BAB7087, and BAB7292, was isolated from blood according to standard procedures. For the genome-wide methylation assay controls, we used samples from subjects previously diagnosed as carriers of maternal uniparental disomy 14 (UPD(14)mat): BAB489 [26], BAB7704, BAB7705, and paternal uniparental disomy 14 (UPD(14)pat): BAB7706 [29]. DNA was extracted from either blood (BAB489) or lymphoblastoid cell lines (BAB7704, BAB7705, and BAB7706).

### Determining triplication size and genomic intervals of absence of heterozygosity (AOH)

Structural variation and AOH in subject BAB7004 were identified by clinical chromosome microarray analysis (CMA) using V10.1 OLIGO [28] which is a custom-designed array with approximately 400,000 interrogating oligonucleotides that include 60,000 probes used for assaying single-nucleotide polymorphisms (SNPs) (Agilent Technologies, Inc., Santa Clara, CA, USA) [28]. To confirm the presence of the AOH segment, as well as to access the origin of triplicated genomic segments, samples were further submitted for SNP array analysis. DNA samples from all family members. Subjects BAB7004, BAB7088, BAB7291, BAB7085, BAB7086, BAB7087, and BAB7292 were hybridized on a HumanOmniExpress-24 Beadchip at the Human Genome Sequencing Center of Baylor College of Medicine. Basic quality control and analysis of the genotyping data were performed using GenomeStudio software (Illumina, Inc., San Diego, CA, USA). B-allele frequency, BAF is calculated as: *B* / (*A* + *B*), where A is the signal of the A allele and B is the signal of the B allele. We further identified AOH segments from unphased exome-sequencing (ES) data using BAM files and preprocessing VCFs according to the BafCalculator algorithm described previously [30, 31].

### Refining triplication size and gene content

To refine the breakpoint junctions of the triplication, we designed a high-density custom tiling-path oligonucleotide microarray spanning chromosome 14q using the Agilent SureDesign website (https://earray.chem.agilent.com/suredesign/website). The average coverage was 1 probe per 1000 bp, spanning the following genomic interval (hg19) chr14: 80,000,000–105,000,000 as described [22]. Probe labeling and hybridization were performed according to the manufacturer’s protocols with minor modifications.

### Oxford Nanopore long read sequencing

#### Sample preparation, sequencing, and base-calling

For this patient sample, two parallel libraries were generated, one using sheared DNA and one using “full-length” DNA. Both libraries were generated from 5-μg genomic DNA isolated from blood. The sheared library was generated by centrifugation in a g-TUBE (Covaris part number 520079) at 1086 rcf. Library preparation was performed in parallel for both sheared and unsheared samples using Oxford Nanopore Technologies’ standard ligation sequencing kit, SQK-LSK109, using 1 μg of the appropriate genomic DNA (gDNA) and the standard protocol with a slight modification in that the final elution was done in 24 μl elution buffer instead of the standard 12 μl. The 24 μl of library was combined with 75 μl sequencing buffer and 51 μl of loading beads and run on a PromethION Beta with PromethION flow cell version 3 using MinKNOW software version 1.11.9 for 64 h. Base-calling was performed using Guppy version 1.4.0 and using a manually set chunk size of 10 kb.

#### Analysis of long-read sequencing data

Alignment of the reads for samples was performed with ngmlr (version 0.2.7) [32] using default parameters along with the –bam-fix parameter for long cigar string support. Structural variant calling was performed with the Sniffles structural variant caller (version 1.0.8) [32], using the following parameter set, chosen to maximize sensitivity “--min_support 1 --minmapping_qual 1 --num_reads_report -1 --genotype”. Suspected structural variants (SV) were confirmed as a concordance between changes in the read depth and the breakpoints for SV events. Upon identification of suspected causal rearrangements, the read names supporting the putative variant were pulled from the sniffles vcf file, and the full collection of mappings for that read were pulled from the bam file, in order to confirm the breakpoint manually in IGV [33], followed by PCR and Sanger sequencing. Sequencing depth was calculated on the resulting alignments using mosdepth (version 0.2.3) [34] with the following parameter set “-F 3588 -Q 1” which calculates coverage depth in 100 bp bins and includes only primary and supplemental alignments. Sniffles and mosdepth were run on the pooled combination of both sheared and unsheared library preparation strategies to maximize the probability of getting reads that unambiguously resolve the breakpoint junctions. Read depth coverage in 100 bp bins was plotted using R ggplot2 package in this region among relative copy-number states (normal, triplication, and duplication regions). The median value of read depth coverage was reported for each copy-number state within the breakpoint junctions that had been mapped to the nucleotide level.

#### Methylation analysis

Nanopore reads were first aligned to GRCh37 using minimap2 v2.15 [35] using the options “-ax map-ont”. Read alignments overlapping MEG3-DMR (chr14: 101,290,524-101,293,978) were extracted, and the reads were assigned to haplotypes using marginPhase v1.0.0 with the supplied model “params.nanopore.json”. For reads from each haplotype assignment, methylation status of CpG sites was assessed using nanopolish call-methylation [36]. Log-likelihood ratios were produced for CpG sites on each read, where positive and negative values indicate likely methylation and nonmethylation, respectively.

### Long-range PCR amplification and breakpoint junction validation

Reverse and forward primer pairs (relative to the reference genome) were designed at the apparent boundaries of the copy-number gain segments as defined by high-density aCGH analysis for triplications and by mapping of the transition interval from copy-number neutral to copy-number gain positions [10]. Long-range PCR was performed using TaKaRa LA *Taq* (Clontech, Mountain View, CA, USA). PCR sample-specific products were sequenced by the Sanger di-deoxy sequencing methodology. PCR and sequencing results were independently repeated to confirm results. Parental DNA was amplified in the same experiments, i.e., used as a negative control and to determine that the breakpoint junction specific PCR was exclusively present in the index subject (i.e., a de novo event). Junction 1 primers are as follows: 7004-trp1F, GGACAGGTAGAGAGCACCCAGTTCC; 7004-dup1F, ACAGCCCCACCACCTTATTTTGAAG. Junction 2 primers: 7004_TRP2NR: TGAAATCAGTGAGTCATGGCTG; 7004_DUP2NR: AAATCAATGAGACTTGGCTGTTC.

### Exome sequencing (ES), variant calling, and selection of de novo and biallelic variants specifying AR disease trait loci affecting chromosome 14

ES was performed at the Human Genome Sequencing Center (HGSC) at Baylor College of Medicine through the Baylor-Hopkins Center for Mendelian Genomics initiative. Using 0.5 μg of DNA an Illumina paired-end pre-capture library was constructed according to the manufacturer’s protocol (*Illumina Multiplexing_SamplePrep_Guide_1005361_D*) with modifications as described in the BCM-HGSC protocol (https://www.hgsc.bcm.edu/content/protocols-sequencing-library-construction). Six pre-captured libraries were pooled and then hybridized in solution to the HGSC VCRome 2.1 design [37] (42 Mb NimbleGen, Cat. No. 06266380001) according to the manufacturer’s protocol *NimbleGen SeqCap EZ Exome Library SR User’s Guide* with minor revisions. The sequencing run was performed in paired-end mode using the Illumina HiSeq 2000 platform, with sequencing-by-synthesis reactions extended for 101 cycles from each end and an additional seven cycles for the index read. With a sequencing yield of 6.6 Gb, the samples achieved 95% of the targeted exome bases covered to a depth of 20X or greater. Illumina sequence analysis was performed using the HGSC Mercury analysis pipeline [38, 39] (https://www.hgsc.bcm.edu/software/mercury) which moves data through various analysis tools from the initial sequence generation on the instrument to annotated variant calls (SNVs and intra-read in/dels). De novo variants were identified in silico by subtracting variants observed in either parent (DNM-Finder; https://github.com/BCM-Lupskilab/DNM-Finder) [40]. Candidate variants/mutations were filtered against publicly available databases including the 1000 Genomes Project, NHBLI GO Exome Sequencing Project (ESP), the Atherosclerosis Risk in Communities Study (ARIC) database, and our in-house generated database of greater than 11,000 exomes. Recessive disease trait specifying biallelic variants affecting disease-associated genes mapping to human chromosome 14 was selected for Sanger di-deoxy DNA sequencing confirmation and to evaluate the transmission genetics segregation pattern in affected and unaffected family members. Prediction scores from four distinct computational algorithms were used to assess pathogenicity of rare variants: PolyPhen-2 [41], SIFT [42], LRT [43] and MutationTaster [44], combined annotation-dependent depletion (CADD) [45]. Copy-number variants (CNVs) using ES were called using XHMM [46].

### Variant confirmation

Potential disease-associated variants identified via ES were verified and evaluated for co-segregation with the clinically observed phenotype using standard PCR amplification. PCR products were purified using ExoSAP-IT (Affymetrix) and sequenced using di-deoxy nucleotide Sanger sequencing at the DNA sequencing core at BCM.

### Chromosome 14 methylation profiling

The specific chromosome 14 methylation profile was determined by analyzing the methylation profile across CpG sites included in the Infinium Human Methylation450 Beadchip (Illumina). DNA samples studied included all family HOU2583 members (affected and unaffected family members) for family-based genomic analysis, samples previously known to carry UPD(14)mat or UPD(14)pat were used as controls. Data analysis was performed using the methylation module of the GenomeStudio Software 2011.1 (Illumina).

DNA samples were first subject to bisulphite conversion using the EZ-96 DNA Methylation kit (Zymo Research Corp., Irvine, California, USA). Bisulfite converted DNA was then profiled using the Infinium HumanMethylation450 Beadarray (Illumina) according to the manufacturer’s instructions. The resulting genome-wide array intensity files (iDat) were imported into the methylation module (version 1.9.0) of GenomeStudio (v2011.1, Illumina) with background normalization and used to generate methylation beta-values for each of the ~ 450,000 loci/sites interrogated on the array. All individuals had less than 5% missing data across all probes. Probes with a detection *p* value > 0.0001 were removed. Our primary experimental goal with this assay was to observe large differences in methylation on chromosome 14 corresponding to imprinted sites; therefore, we did not employ further normalization for color balance or channel, or tissue deconvolution of samples prior to analysis. Probes assigned to chromosome 14 on the accompanying 450 K manifest were filtered to remove low-intensity probes, exported as a data table to RStudio (v 1.0.143), and analyzed using R (v3.1.3). Downstream analyses involved assigning the resulting *β* values (i.e., proportion of methylated cytosines) at a given CpG probe site as follows: *β* > 0.8 methylated, 0.2 > *β* < 0.8 partially methylated, and *β* < 0.2 unmethylated. An in-house methylation visualization app, MethylViz, developed using ShinyR, was used to visualize methylation values across chromosome 14 and at the regions of interest (code available upon request).

## Results

### Clinical spectrum

The male proband (BAB7004) is presently a 22-year-old. His clinical phenotype includes mild to moderate intellectual disability (ID), short stature (< 5%), apparent microcephaly (< 25%), and mild facial dysmorphisms. He was initially evaluated at 4 years of age due to short stature and speech delay, with a vocabulary of 10–20 single words. In retrospect, he was born at about 39 weeks gestation and weighed 2.6 kg (3rd centile). His growth and development were slow. He walked at 1.5 years. He had a broad nasal root, frontal bossing, anteverted nares, long eyelashes, high palate, micrognathia, mild *pectus excavatum*, brachydactyly with 2-3-4 partial toe syndactyly, hypermobility of joints, mild hypotonia, undescended testes, *pes planus* and small hands and feet. Blood chromosome analysis, echocardiogram and brain MRI were all normal. He returned for evaluation of mild to moderate ID and short stature. His physical exam at 22 years of age was similar to the findings listed above. However, he now had descended testes without surgical intervention. He now spoke in full sentences with articulation errors. Puberty had occurred at 16 years of age, and he was otherwise generally healthy. In addition to the proband, there are four healthy and developmentally normal siblings. The father is 178 cm and mother is 167.5 cm, conferring a mid-parental height of about 179 cm for male offspring (63th centile for an adult male). Physical examination of the proband revealed height of 150 cm (− 3.6 SD), weight 68 kg (50th centile) and occipital frontal circumference OFC 54 cm (25th centile).

### Whole genome sequencing

A genomic DNA sample from individual BAB7004 was subjected to nanopore sequencing using two parallel libraries. The sheared DNA ONLL03750 library yielded a total of 51.7 Gbp of genome sequence with 12,168,534 quality passed reads and a read N50 of 12,625 bp. About 64.14% of reads mapped to the hg38 haploid human reference genome. Of these, 224,165 reads mapped to chromosome 14 and 217,263 mapped to chromosome X. The mean coverage for this library of chr14 was 12.5x and for chrX was 5.9x. The full-length DNA ONLL03755 library yielded a total of 40.14 Gbp with 10,544,515 quality passed reads with a read N50 of 10,504 bp. About 40.31% of the reads mapped to hg38 reference genome. Of those, 113,682 reads mapped to chromosome 14 and 108,428 mapped to chromosome X. The mean coverage for the full-length ONLL03755 library of chr14 was 3.4x and for chrX was 1.6x. The mean coverage for the whole genome for ONLL03750 was 11.2x and for ONLL03755 was 3.0x.

### Characterization of triplication revealed a DUP-TRP/INV-DUP structure

**Fig. 1:**
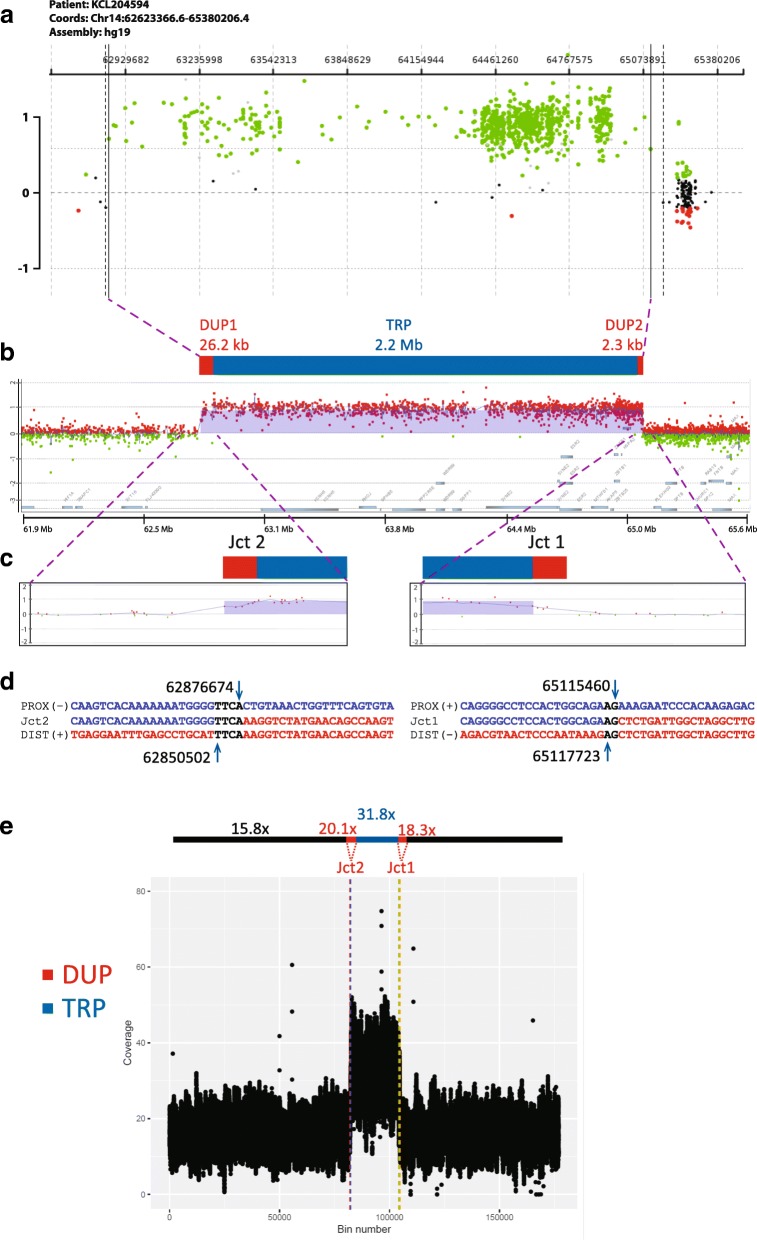
Triplication followed by ROH shows DUP-TRP/INV-DUP pattern. **a** Agilent aCGH (V10.1) plot of chromosome 14q23.2q23.3 region harboring a 2.2-Mb triplication (TRP; log2 ratio 1). **b** Agilent tiling high-density aCGH revealed a triplication (TRP; blue box) flanked by small duplications (DUP; red boxes) at both centromeric (Jct2) and telomeric junctions (Jct1) (**c**). **d** Color-matched sequence alignment of Jct1 and Jct2. Sequencing data of long-range PCR product was obtained by standard Sanger. Structure is now defined as DUP-TRP/INV-DUP. Genomic reference segments are displayed in blue (PROX, proximal) and red (DIST, distal). Strand orientation is indicated in parentheses. Microhomology at the junction is represented in black. **e** Read depth coverage plot of 17.7  Mb segment of 14q (chr14: 54,179,300-71,874,900) that includes the DUP-TRP/INV-DUP region. Normalized read depth coverage confirms the relative copy-number differences along the CGR consistent with a triplication (31.8/15.8 = 2) flanked by duplications (DUP1 = 20.1/15.8 = 1.3; DUP2 = 18.3/15.8 = 1.2). *Y*-axis: read depth coverage; *X*-axis: 100 bp bins at 14q. Genomic coordinates are in hg19

A de novo copy-number gain spanning ~ 2.2 Mb consistent with heterozygous triplication (locus CN = 4) involving cytogenetic subbands 14q23.2q23.3 followed by 42.2 Mb of copy-number neutral runs of homozygosity (ROH)/absence of heterozygosity (AOH) spanning from 14q23.3q32.33 was identified in patient BAB7004 by a genome-wide custom-designed clinical chromosome microarray analysis (CMA) [27, 28](data not shown). Custom high-resolution aCGH further revealed a complex genomic rearrangement (CGR) consisting of a large triplication (2.2 Mb) flanked by small duplications at both centromeric (26.2 kb) and telomeric sides (2.3 kb) (Fig. 1). ES CNV calls were also analyzed along with the aCGH data to independently confirm the presence of a copy-number gain at chromosome 14 as well as to rule out any additional CNV that could have contributed to the genotype. ES analysis confirmed a copy-number gain call of 1.88 Mb from chr14: 63,174,225-65,056,057 (hg19), which overlaps with the aCGH detected CNV of 2.2 Mb. No additional potential pathogenic CNVs were identified in the ES data by XHMM. Using BafCalculator [30, 31] on unphased ES data, and a comparison to genome-wide SNP array data, revealed a similar-sized ROH/AOH copy-number neutral genomic interval mapping to chromosome 14.

Inverted triplications flanked by duplications is a CGR pattern referred to as DUP-TRP/INV-DUP [10] in which the triplicated segment is inserted in an inverted orientation amid two duplicated copies with respect to the reference genome. We tested for this hypothesized CGR structure by designing PCR outward-oriented primers at apparent boundaries, as evidenced by change in copy-number states, of the copy-number gain segments (DUP-TRP or TRP-DUP), i.e., breakpoint junctions Jct1 and Jct2, as defined in Fig. 1. A single PCR band was obtained exclusively in the BAB7004 DNA sample; further Sanger sequencing confirmed that the triplicated segment is inverted, consistent with a DUP-TRP/INV-DUP structure. Jct2 could not be obtained using this PCR junction amplification and Sanger sequencing approach, but it was further mapped by Nanopore long reads and validated by Sanger sequencing. Sequencing alignments are shown in Fig. 1d. There is a 2 bp and 4 bp of microhomology at Jct1 and Jct2, respectively; no additional complexity such as insertions, de novo single-nucleotide variants (SNVs) or indels were observed flanking ~ 150 bp of these sequence determined junctions [22, 47, 48].

Nanopore long-read sequencing, using algorithms that incorporate both split-reads and read depth data, supports the structure proposed for DUP-TRP/INV-DUP (Fig. 1e). Transition between copy-number neutral segments (15.8x) to flanking small duplications (26.2 kb and 2.3 kb) to the large triplication (2.2 Mb) was possible using normalized read depth coverage values from 100 bp bins calculated within the defined duplication junctions; Jct2 (chr14: 62,850,502-62,534,442) that defines flanking DUP1 (20.1x), Jct1 (chr14: 65,115,460 65,117,723) that defines DUP2 (18.3x), and (chr14: 62,876,674-65,115,460) that defines TRP (31.8x).

The relative normalized read depth coverage enables distinction among neutral copy number (*N* = 2), duplications (expected relative CN = 1.5; observed relative CN for DUP1 = 1.3 and for DUP2 = 1.2), and triplication (expected relative CN = 2.0; observed relative CN for TRP = 2.0) (Fig. 1e). The small size of flanking duplications (26 kb and 2.3 kb) compared to the large size of the triplication (2.2 Mb) likely contributes to both the absence of detection and resolution of the DUP1 and DUP2 by clinical CMA and the higher precision of the observed copy-number observation for long-read sequencing analysis for the TRP versus DUP.

### Copy-number neutral ROH/AOH segment is due to perturbed transmission genetics and a segmental maternal uniparental disomy

**Fig. 2:**
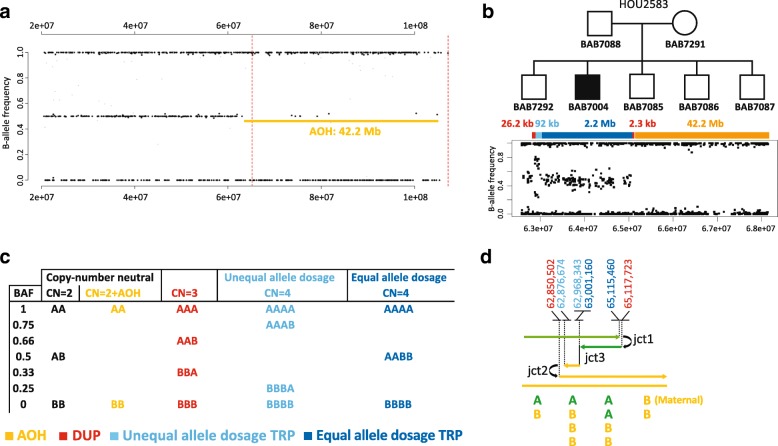
SNP analysis enabled AOH/ROH detection in addition to a copy-number neutral junction supporting underlying replication-based mechanism. **a** Agilent aCGH (V10.1) SNP array data (B-allele frequency, BAF) for chromosome 14 indicates a 42.2 Mb of AOH. **b** Top: pedigree structure of family HOU2583; Bottom: Illumina SNP array HumanOmniExpress-24 Beadchip B-allele frequency (BAF) plot of ~ 42 Mb telomeric segment spanning 14q (chr14:62584057-107287663) reveals a de novo complex genomic rearrangement (CGR) and ROH/AOH exclusively present in BAB7004 (please refer to Additional file 1: Figure S1 for SNP array results in other family members). BAF revealed that the TRP segment presents two distinct genotypes: a small one (92 kb) with unequal allele dosage, followed by a larger one with equal allele dosage (2.2 Mb). Unequal allele dosage TRP, light blue rectangle; equal allele dosage TRP, dark blue rectangle; flanking small DUP, red rectangle; AOH, orange rectangle. **c** Expected BAF genotypes for distinct copy-number states. **d** Color-matched schematic model of the 14q23.2q23.3 CGR formation in BAB7004. CGR presents at least three breakpoint junctions, i.e. Jct1, Jct2, Jct3, which are hypothesized to be generated by template switches during replication-based repair (see main text for discussion). Top: genomic coordinates of junctions inferred from multiple technical approaches. Bottom: representation of the SNP allele dosage in each segment involved in this CGR. A, B: SNP alleles. Genomic coordinates are in hg19

The triplication gain was again confirmed by SNP array. ROH/AOH spanning 42.2 Mb of 14q that extends from the end of the triplicated segment until the telomere was identified in the diagnostic laboratory by CMA (Fig. 2a). We further confirmed the size of ROH, equivalent to regions of AOH, and genomic content by SNP array HumanOmniExpress-24 Beadchip (Fig. 2b) and ES analysis of AOH regions using BafCalculator. Both DUP-TRP/INV-DUP and AOH constitute a CGR that occurred de novo in individual BAB7004, and it is not present in his parents or unaffected siblings (Fig. 2b and Additional file 1: Figure S1). SNP array revealed maternal inheritance of all SNPs in the AOH region indicating a segmental maternal isodisomy, and specifically UPD(14)mat. By calling SNVs on the nanopore generated data and calculating allele frequencies, the presence and extent of the ROH/AOH could be independently corroborated.

### Triplication is formed by two segments with distinct genotypes

Inspection of B-allele frequencies of the DUP-TRP/INV-DUP segments revealed that the triplication was constituted by two distinct allele genotypes: a small, ~ 92 kb, region presenting SNPs with unequal parental allele contribution, i.e., ABBB or BAAA followed by a large segment of ~ 2.2 Mb with equal parental allele dosage, i.e., AABB (Fig. 2b, c). No copy-number differences exist between the two genotype states. Therefore, this result indicates that distinct parental chromosomal segments from homologous chromosomes were joined together without copy-number alteration. Such a transition site, arbitrarily named breakpoint junction 3 (Jct3), was previously observed in two out of five cases in our published cohort of triplications followed by AOH [22]. A model for the formation of this CGR in patient BAB7004 is shown in Fig. 2d. Flanking duplications of 26, 2 kb and 2.3 kb were not detected by the high-resolution SNP array.

### Variants present in triplicated and copy-number neutral ROH/AOH segments unlikely to contribute to the clinical phenotype

We performed trio ES to access the presence of rare and/or potentially pathogenic variants in homozygosis within the 2.2-Mb triplicated region and the 42.2-Mb copy-number neutral ROH/AOH genomic interval of chromosome 14 that may have potentially contributed to the clinical phenotype of patient BAB7004. Two rare missense variants affecting disease-associated genes, *SYNE2* (NM_015180), chr14:g.64469886A>G (c.A4235G; p.E1412G) and *POMT2* (NM_013382), and chr14:g.77744801A>T (c.T2083A; p.W695R) were detected and independently confirmed by Sanger sequencing. Heterozygous variants affecting *SYNE2* cause autosomal dominant Emery-Dreifuss muscular dystrophy 5 (MIM#612999). *SYNE2* is within the triplicated segment; therefore, it is present in four copies. Since it maps to the equal dosage allele triplicated segment, reference and variant alleles are present two times each. The variant allele was inherited from the heterozygous mother, BAB7291 (Additional file 1: Figure S2a). In addition, this allele is not present in any of the following databases: our in-house database consisting of ~ 9000 exomes with a wide variety of Mendelian phenotypes; the Atherosclerosis Risk in Communities Study (ARIC) database of ~ 11,000 exomes, dbSNP, or ExAC; and gnomAD databases. The variant affecting *POMT2* was inherited from the heterozygous mother but it is present in the homozygous state in BAB7004 as it maps within the AOH region (Additional file 1: Figure S2b). However, although the subject presents with hypotonia, he does not have features of muscular dystrophy and does not have severe intellectual disability as can be observed in individuals with *POMT2* pathogenic alleles. Therefore, in this clinical context, the homozygous variant is unlikely to contribute to his clinical phenotype.

Genome-wide analysis of de novo variants revealed a missense affecting the beta-tubulin class IIA gene, *TUBB2A*, (NM_001069), chr14: g. 3154999C>T (c.G436A, p.G146R). Importantly, part of *TUBB2A* overlaps with four distinct segmental duplications which includes the exon on which such de novo variant maps. One of the segmental duplications consists of *TUBB2B*, which shares > 99% identical nucleotides and localizes ~ 66 kb distal to *TUBB2A* in a direct orientation. To further validate the variant, we designed a primer pair to specifically amplify only the *TUBB2A* segment and not the other paralog sequences (Forward: 5′ GCAAAACTGAGCACCATAGTT 3′ and Reverse: 5′ CCCAGTGTTTTTGAGGTCACTG 3′, amplicon size: 1327 bp). Standard Sanger sequencing confirmed the presence of the variant (Additional file 1: Figure S2c).

### Chromosome 14 methylation profile is consistent with maternal imprinting

**Fig. 3:**
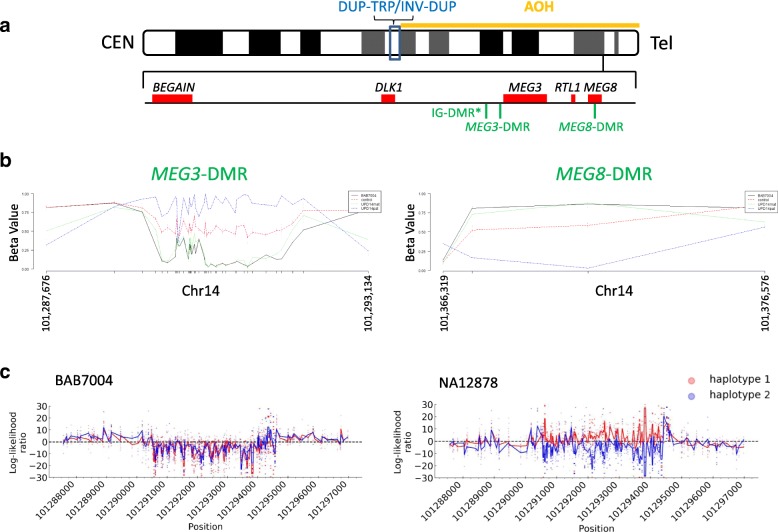
Methylation profile consistent with maternal imprinting in UPD region. **a** Ideogram of chromosome 14q displays location of DUP-TRP/INV-DUP and AOH segment (top). Two out of three differential methylated regions (DMR) located at 14q32.2 (bottom) were assayed by Infinium Human Methylation450 Beadchip (Illumina). *DMR not assayed. **b** Beta value (β, i.e., percentage of methylation of a given cytosine) plots: *β* > 0.8 methylated, 0.2 > *β* < 0.8 partially methylated, *β* < 0.2 unmethylated. *X*-axis indicates genomic coordinates for each CpG dinucleotide assayed by the array for *MEG3*-DMR and *MEG8*-DMR (hg19). Color-matched dashed lines display *β* values for three groups of assayed individuals: blue, UPD(14)pat individuals (BAB7706); green, UPD(14)mat individuals (BAB489, BAB7704, BAB7705); red, controls without UPD(14) (BAB7085, BAB7086, BAB7087, BAB7088, BAB7291, BAB7292). Black line displays BAB7004 results. In both *MEG3*-DMR and *MEG8*-DMR, BAB7004 displays methylation profile consistent with UPD(14)mat. **c** Plot displays the methylation log-likelihood ratio (LLR) for the CpGs spanning the *MEG3*-DMR locus calculated using Nanopolish [36] in BAB7004 and control NA12878. Positive LLR indicates methylated CpG. Haplotypes are color-matched. For BAB7004, both haplotype alleles show skewed lack of methylation across *MEG3*-DMR consistent with maternal imprinting whereas control NA12878 shows a haplotype pattern expected for a non-UPD sample. Genomic coordinates are in hg19

Chromosome 14 is subjected to imprinting; at least three differential methylation loci are known: IG-DMR and *MEG3*-DMR, both of which are hypermethylated in the paternal chromosome whereas hypomethylated in the maternal chromosome [49, 50] in addition to the recently described *MEG8*-DMR which is hypermethylated in the maternal chromosome [51] (Fig. 3a). Infinium Human Methylation450 Beadchip has probes mapping to the CpG sites of *MEG3*-DMR and *MEG8*-DMR both of which display methylation patterns consistent with maternal imprinting in BAB7004 (Fig. 3b). Finally, for the *MEG3*-DMR in BAB7004, we also used the nanopore sequencing reads for calling the base methylcytosine as one indication/readout of methylation status which confirmed the hypomethylation status of that locus compared to a non-UPD sample control (Fig. 3c).

## Discussion

De novo TRP/AOH has been reported involving distinct chromosomes including 1, 5, 6, 9, 10, 11, 14, and 22 [20–24, 52], in individuals presenting diverse phenotypes and congenital malformations. Here, we describe a family with a patient who carries a CGR, TRP/AOH, which is a rare combination of a CNV constituted by a 2.2 Mb triplication plus a 42.2 Mb segmental maternal isodisomy wherein the latter includes the imprinted locus at the 14q32 region. Each one of the patients carrying the TRP/AOH reported thus far has a distinct segment of the chromosome affected (for instance, 1p36.12, 6q24.1, 10q26.13, 14q32.12, 14q23.3q32.33, 9q12-q21.11) [22], therefore indicating that the underlying mechanism is not locus specific. Involvement of both parental chromosomes in the origin of triplicated segments indicates that such rearrangements are post-zygotic, likely occurring very early during development as no evidence of mosaicism was observed in the patients reported thus far [20, 21, 23] - potentially a perizygotic SV mutagenesis event [53]. In the subject reported here, analysis of both secondary somatically-imprinted DMR sites in 14q32, *MEG3*-DMR and *MEG8*-DMR, confirmed maternal specific imprinting patterns which supports the hypothesis that the TRP/AOH occurred very early in development, likely before the blastocyst stage [50]. This unique family provides a compelling ‘experiment of nature’ in which one might dissect phenotypic consequence of trisomy 14 due to gene dosage effects (due to triplication or duplication) versus copy-number neutral chromosome 14 locus imprinting and transmission genetics reduction to homozygosity by ROH/AOH.

CGR breakpoint junction analysis by customized aCGH in the study subject, as well as patients published previously [22], revealed that the triplications are actually flanked by small duplications, the latter is often not detected by lower resolution clinical array CGH or CMA [22]. In addition, the triplications are inverted in all cases for which the orientation of the copy-number gain segments were studied [20, 21, 23, 24]. Molecular characterization unveiled that the currently studied family and patient ascertained TRP/AOH structure actually represents the same DUP-TRP/INV-DUP pattern described previously [10, 48] for triplications affecting diverse disease-associated loci genome-wide [54].

Here, we have used long-read single-molecule sequencing which independently confirms the DUP-TRP/INV-DUP structure previously proposed, i.e., an inverted triplication flanked by duplications, and enabled assessment of methylated bases at specific loci. The results of long-read genomic sequencing allowed read depth determinations to be used as a surrogate measure of relative copy number deriving an apparent “DUP-TRP-DUP” type of CGR structure as seen in a high-resolution array. Such a complex structure is formed by a microhomology-mediated break-induced replication (MMBIR) mechanism with at least two template switches that result in two breakpoint junctions, Jct1 and Jct2, corresponding to the insertion of the inverted triplication. Of note, a 2-bp and 4-bp microhomology were observed at Jct1 and Jct2, respectively, consistent with a microhomology -mediated template switch (TS). These “breakpoint junctions” can perhaps more accurately be thought of as “join-points” wherein a template switch, during MMBIR driven CGR formation, “joins” discontinuous sequence from the reference haploid genome together, creating an apparent breakpoint junction [22, 25, 54, 55]. Intriguingly, autosomal DUP-TRP/INV-DUP, as in the one reported here and others elsewhere [22], can be generated by TS between chromosome homologs, i.e., interchromosomal versus intrachromosomal recombination, in contrast to X-linked DUP-TRP/INV-DUP which are apparently preferentially generated by intrachromosomal TSs [10, 47]. Moreover, some of the autosomal DUP-TRP/INV-DUP are constituted by two distinct allele genotypes on which the transition between these genotypes constitutes a novel type of junction (Jct3) formed by template switches between homologous chromosomes without an accompanying copy-number alteration [22]. Such copy-number neutral junction, Jct3, also observed in this present CGR, has been hypothesized to be formed by template-switching between homologous chromosomes involving allelic rather than non-allelic segments, likely by a break-induced replication (BIR) mechanism of recombination [56], consistent with a copy-number neutral junction formation [22].

Each one of the three cases with evidence informative for a third template switch reported thus far involve a distinct chromosome, chromosome 14 in BAB7004 (described herein), chromosome 1 in DECIPHER_257814 and chromosome 10 in BAB3923 [22], but a similar pattern starts to emerge. For example: (i) all three cases are characterized as DUP-TRP/INV-DUP followed by AOH; (ii) the unequal parental allele contribution segment is small, varying in size from 126 kb to 150 kb, whereas the equal parental allele contribution segments are large varying from 2.2 Mb to 8.7 Mb; (iii) the unequal parental allele dosage segment maps close to Jct2 which is the transition junction from DUP-TRP/INV-DUP structure to AOH (Fig. 2d).

Maternal UPD(14) was first reported in the early 1990s: the syndrome as a clinical entity was reported by I.K.Temple and colleagues [57] for heterodisomy and by Liu et al. [26] for isodisomy. It is characterized by pre- and postnatal growth retardation, relative macrocephaly, hypotonia and motor delay, feeding problems early in life, truncal obesity, precocious puberty in both males and females, and facial characteristics including a broad forehead and fleshy nasal tip [58, 59]. Maternal UPD(14) syndrome presents overlapping clinical features with Prader-Willi (PWS, OMIM#176270) [60] and Russel-Silver syndrome (SRS, OMIM#180860) [61]. Molecular causes vary: a recent review of published cases culled from the literature [59] indicates that ~ 78% of reported cases result from full isodisomy or heterodisomy UPD(14)mat, whereas paternal deletions on chromosome 14 spanning the 14q32 locus and loss of methylation at the intergenic DMR (IG-DMR) contribute to ~ 10% and ~ 12% of the molecular causes, respectively. Relevant for this study, segmental UPD14 has been very rarely reported in the literature [62]. Genotype-phenotype correlation in patients with a deletion affecting the paternal allele of chromosome 14 was important to delineate the imprinted genomic region [58]. In fact, Kagami and colleagues used such patients to narrow the region associated with maternal UPD(14) syndrome to 108 kb that includes imprinted genes *DLK1* and *GTL2/MEG3* as well as an important cis-regulatory DMR, IG-DMR [49].

In general, the patient presented here presents a clinical picture that overlaps significantly with the majority of subjects with UPD(14)mat [59], including short stature, small hands and feet, hypotonia, somewhat lax joints, and developmental delay; parents reported that he did not have early onset of puberty. Early puberty has been reported in 87% of patients with UPD(14)mat [59], 100% of patients carrying loss of methylation in the paternal germline IG-DMR at 14q32, and 2/3 of the patients carrying deletion of the paternal 14q32. It should be noted, however, that some cases in the literature reported as “precocious” or “early” puberty do not meet standard clinical definitions and criteria for “precocious puberty”. In contrast, this precocious puberty is mostly absent in partial maternal or paternal trisomy 14q [63], in deletions involving 14q32 from maternal origin [64], which indicates that early-onset puberty is triggered by the lack of expression of one of the paternally imprinted genes mapping within the 14q32 [59]. Consistent with this hypothesis, partial deletion of the paternally expressed gene *DLK1* was reported in a family with familial central precocious puberty when deletion was inherited from the father although this family shows incomplete penetrance for the trait [65]. Therefore, it is notable that the individual reported here does not have precocious puberty, although he has partial isodisomy UPD(14)mat which is associated with the expected patterns for this genomic finding (Fig. 3). One potential explanation is that the partial tetrasomy of 14q23.2q23.3 compensates for the predicted lack of *DLK1* expression or it changes the expression of the maternal copy which may preclude the phenotype of early-onset puberty. In addition a history of cryptorchidism is a known risk factor for pubertal delay.

The majority of subjects with UPD(14)mat have been reported to have normal intellectual development, although measured IQ, when available, has often been in the low normal range [59]. BAB7004 presents with intellectual disability and speech delay which may still be considered within the spectrum of what is generally reported in Temple syndrome [66]. The identified triplicated segment in subject 7004 spanning 14q23.2q23.3 does not have a known dosage-sensitive gene associated with ID/DD within its 2.2 Mb interval. Partial trisomy of chromosome 14q is an extremely rare condition [63, 67], with extensive clinical variability which is consistent with the genomic content and size variability of the higher copy-number region involved in the cases reported [63].

The majority of individuals with trisomy 14 have craniofacial abnormalities, intellectual disabilities, neuromotor delay, and postnatal growth retardation. Very few individuals with trisomy 14 are reported with partial trisomy that overlaps the triplicated segment in the patient described here; however, we can not rule out a gene dosage effect either from direct alteration of the genomic copy number or disruption of regulatory regions, or topologically associated domains (TAD) for genes nearby the CNV. Of note, individuals with trisomy 14 mosaicism have a distinct and recognizable phenotype that shares little in common with the phenotype of UPD14 [68].

The phenotype of the UPD cases can also potentially be modulated or expanded by the contribution of expressed recessive disease traits. We investigated this possibility by performing trio ES analysis to search for homozygous pathogenic rare variants affecting known disease genes that map within the long-arm of chromosome 14. Within that segment, we found a rare homozygous missense mutation, c.T2083A:p.W695R, in *POMT2* located within the maternally inherited UPD region. POMT2 is an endoplasmic reticulum membrane protein that, together with POMT1, catalyzes the first step in the synthesis of the O-mannosyl glycan. This variant is present at a very low frequency in both dbSNP and ExAC (~ 0.015%) as well as gnomAD (0.009%) and none of those variant databases report homozygous individuals. Pathogenic variants in *POMT2* can cause one of three types of recessive muscular dystrophy-dystroglycanopathy: congenital with brain and eye anomalies, type A, 2 (MDDGA2, MIM#613150); congenital with mental retardation, type B, 2 (MDDGB2, MIM#613156); and limb-girdle, type C, 2 (MDDGC2, MIM#613158). Although it is possible that the lower limb hypotonia and the intellectual disability found in this patient are caused by the biallelic variant allele in *POMT2*, this subject does not have features of muscular dystrophy; therefore, we consider this an unlikely possibility.

In aggregate, these data provide experimental evidence that, in humans, triplication generated post-zygotically can lead to segmental UPD accompanied by an abnormal methylation pattern of specific loci mapping to chromosome 14. Furthermore, the impact on the disease phenotype resulting from the underlying replication-based mutational mechanism in humans can extend beyond that due to the formation of copy-number variants and recessive trait manifestations when only one parent is a carrier. In this study, we apodictically show traits associated with imprinted loci can manifest.

## Conclusions

In summary, we report a 22-year-old male with segmental maternal uniparental isodisomy of chromosome 14 along with a DUP-TRP/INV-DUP that occurred post-zygotically by a template-switching replicative repair mechanism, most likely MMBIR. An apparent interchromosomal template switch results in methylation patterns of DMRs consistent with what is observed in individuals with UPD(14)mat. Therefore, intrachromosomal triplications generated by replication-based mechanisms can result in UPD and imprinting diseases. This case indicates that individuals with segmental UPD should have testing to specifically assess for CNVs that could have arisen post-zygotically as part of the replicative process.

## Additional file

Additional file 1 **Figure S1.** Illumina SNP array HumanOmniExpress-24 Beadchip B-allele frequency (BAF) plots of the telomeric segment spanning 14q (chr14:62584057-107287663) confirms a de novo complex genomic rearrangement (CGR) and ROH/AOH in BAB7004. **Figure S2.** Sanger sequencing and segregation of selected rare SNVs affecting pathogenic genes in BAB7004 detected by trio analysis of ES data. Genomic coordinates are in hg19 (PPTX 1716 kb)

## Abbreviations

aCGH: Array comparative genomic hybridization
AOH: Absence of heterozygosity
BIR: Break-induced replication
CADD: Combined annotation-dependent depletion
CGRs: Complex genomic rearrangements
CNVs: Copy-number variants
DMRs: Differentially methylated regions
DUP-TRP/INV-DUP: Inverted triplication flanked by duplications
FoSTeS: Fork stalling and template switching
MMBIR: Microhomology-mediated break-induced replication
NAHR: Nonallelic homologous recombination
OFC: Occipital frontal circumference
ROH: Runs of homozygosity
SNPs: Single-nucleotide polymorphisms
TRP: Triplications
UPD: Uniparental isodisomy
