# Supplementary material for: Interchromosomal template-switching as a novel molecular mechanism for imprinting perturbations associated with Temple syndrome
Source: Genome Med. 2019 Apr 23;11:25. doi: 10.1186/s13073-019-0633-y (PMC6480824; doi:10.1186/s13073-019-0633-y)
Supplement: Supplementary file 1 — Figure S1. Illumina SNP array HumanOmniExpress-24 Beadchip B-allele frequency (BAF) plots of the telomeric segment spanning 14q (chr14:62584057-107287663) confirms a de novo complex genomic rearrangement (CGR) and ROH/AOH in BAB7004. Figure S2. Sanger sequencing and segregation of selected rare SNVs affecting pathogenic genes in BAB7004 detected by trio analysis of ES data. Genomic coordinates are in hg19 (PPTX 1716 kb) [file 13073_2019_633_MOESM1_ESM.pptx]

## Slide 1
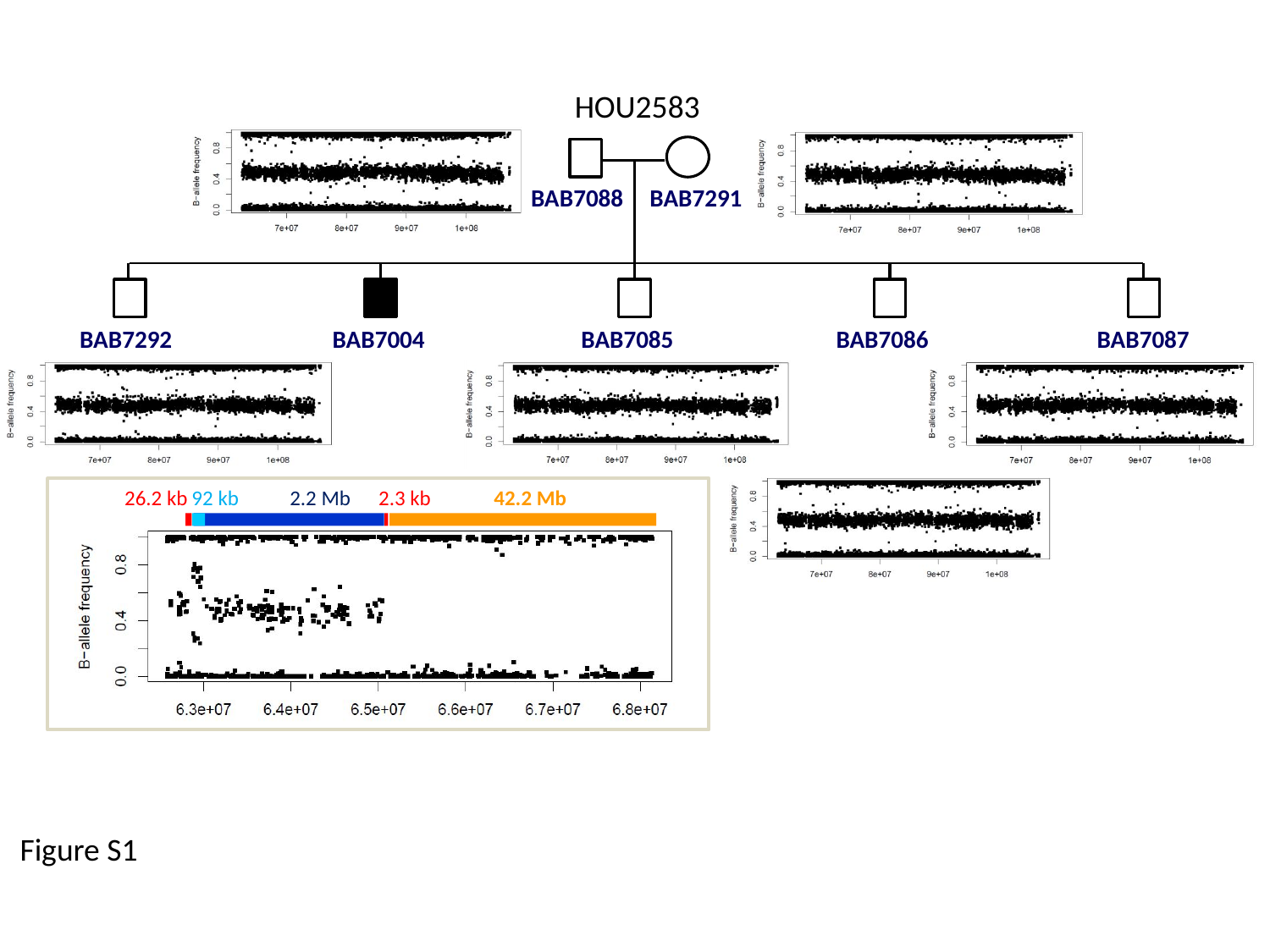

HOU2583
BAB7088
BAB7291
BAB7292
BAB7004
BAB7085
BAB7086
BAB7087
42.2 Mb
26.2 kb
92 kb
2.2 Mb
2.3 kb
Figure S1

## Slide 2
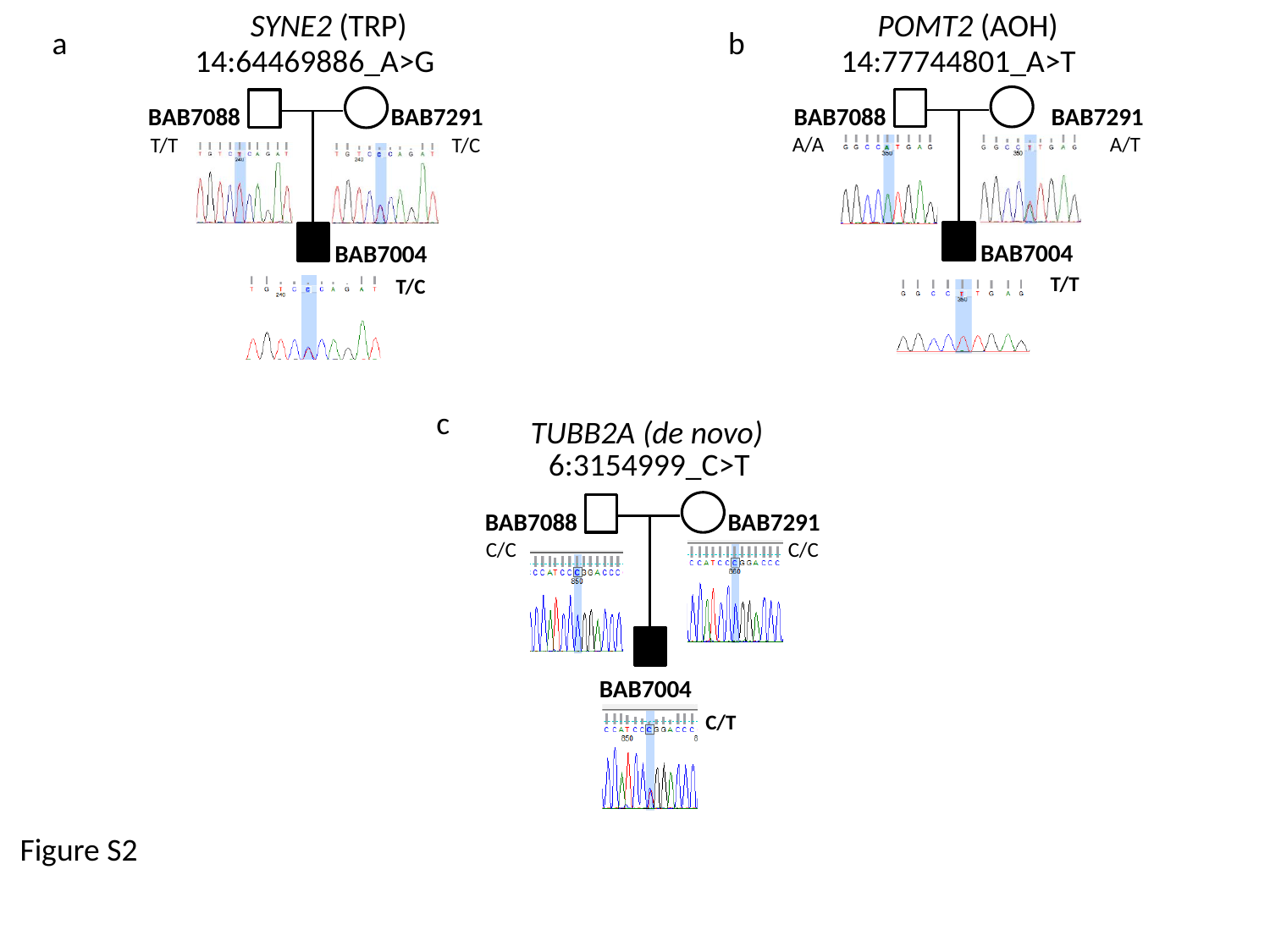

SYNE2 (TRP)
POMT2 (AOH)
a
b
14:77744801_A>T
14:64469886_A>G
BAB7088
BAB7291
BAB7088
BAB7291
A/A
 A/T
T/T
 T/C
BAB7004
BAB7004
 T/T
 T/C
c
TUBB2A (de novo)
6:3154999_C>T
BAB7088
BAB7291
C/C
 C/C
BAB7004
 C/T
Figure S2
